# Smartphone-Based Activity Recognition for Indoor Localization Using a Convolutional Neural Network †

**DOI:** 10.3390/s19030621

**Published:** 2019-02-01

**Authors:** Baoding Zhou, Jun Yang, Qingquan Li

**Affiliations:** 1 College of Civil Engineering, Shenzhen University, Shenzhen 518060, China; bdzhou@szu.edu.cn (B.Z.); yangj256@mail3.sysu.edu.cn (J.Y.); 2 Guangdong Key Laboratory of Urban Informatics, Shenzhen University, Shenzhen 518060, China; 3 Shenzhen Key Laboratory of Spatial Smart Sensing and Services, Shenzhen University, Shenzhen 518060, China; 4 Institute of Urban Smart Transportation & Safty Maintenance, Shenzhen University, Shenzhen 518060, China

**Keywords:** activity recognition, indoor localization, deep learning, smartphone

## Abstract

In the indoor environment, the activity of the pedestrian can reflect some semantic information. These activities can be used as the landmarks for indoor localization. In this paper, we propose a pedestrian activities recognition method based on a convolutional neural network. A new convolutional neural network has been designed to learn the proper features automatically. Experiments show that the proposed method achieves approximately 98% accuracy in about 2 s in identifying nine types of activities, including still, walk, upstairs, up elevator, up escalator, down elevator, down escalator, downstairs and turning. Moreover, we have built a pedestrian activity database, which contains more than 6 GB of data of accelerometers, magnetometers, gyroscopes and barometers collected with various types of smartphones. We will make it public to contribute to academic research.

## 1. Introduction

In the indoor environment, human activity contains rich semantic information, for example, if a user’s activity is recognized as taking an elevator, the location of the user can be inferred to the elevator. These activities can be used as the landmarks for indoor localization and mapping [1,2,3,4,5]. For example, when a user’s activity is detected as “elevator”, his/her location may be at the elevator. The recognition of human activities has been approached in two different ways, namely ambient sensing methods and wearable sensing methods [6]. The ambient sensing methods make use of the devices fixed in predetermined point of interest to sense human activities, such as video camera [7] and WiFi signal [8]. Wearable sensing methods are based on the sensors attached to the user. Recently, with the development of sensor technology, wearable sensing methods have become more and more popular. The wearable sensing methods can be implemented directly in smartphones [9].

Human activities can be categorized into different types [6], including ambulation (e.g., walking, running, sitting, standing still, lying, climbing stairs, descending stairs, riding escalator, and riding elevator), transportation (e.g., riding a bus, cycling, and driving), daily activities (e.g., eating, drinking, working at the PC, etc.), exercise/fitness (rowing, lifting weights, spinning, etc.), military (e.g., crawling, kneeling, situation assessment, etc.), and upper body (Chewing, speaking, swallowing, etc.). In this paper, we focus on the indoor activity recognition, which contains context information and can be used for indoor localization.

Activity recognition using smartphones is a classic multivariate time-series classification problem, which makes use of sensor data and extracts discriminative features from them to recognize activities by a classifier [10]. As we know, time-series data have a strong one-dimensional structure, in which the variables temporally nearby are highly correlated [11]. Traditional methods usually consist of two parts: feature extraction and classification. They rely on extracting complex hand-crafted features which require laborious human intervention and leads to the incapability of pedestrian activities identification. One of the challenges of activity recognition is feature extraction. The activity recognition performance depends highly on the feature representations of the sensor data [10].

Recently, the concept of deep learning has attracted considerable attention. There are numerous applications based on deep learning, such as image processing [12], speech enhancement [13], intelligent transportation system [14], indoor localization [15], and so on. Many studies have confirmed that a deep learning model has a better feature representation capability and, accordingly, could more effectively deal with complexity classification tasks [16]. This is because the deep learning methods fusing feature extraction and classification together with a neural network which can automatically learn proper features.

In this paper, we propose a deep learning-based method for indoor activity recognition by using the combination of data from multiple smartphone built-in sensors. A new convolutional neural network (CNN) has been designed for the one-dimensional sensor data to learn the proper features automatically. Experiments show that the proposed method achieves approximately 98.33% accuracy in identifying nine types of activities, including still, walk, upstairs, up elevator, up escalator, down elevator, down escalator, downstairs and turning. The contribution of this paper is designing a deep learning framework to efficiently recognize activities, which can be used for indoor localization. Moreover, we have built a pedestrian activity database, which contains more than 6 GB of data of accelerometers, magnetometers, gyroscopes and barometers collected with various types of smartphones. We will make it public to contribute to academic research.

The rest of this paper is organized as follows. Section 2 reviews related work. Section 3 presents the proposed method. Section 4 presents the experimental results and analysis. Section 5 concludes the paper.

## 2. Related Works

There are many sensors that can be used for human activity recognition, such as cameras, depth cameras, wireless, inertial sensors, and so on. Also, a large number of methods have been proposed for human activity recognition. This section lists the related works of human activity recognition from two aspects: sensors used and methods used.

### 2.1. Sensors Used for Human Activity Recognition

The sensors used for human activity recognition can be categorized into two types, namely, ambient sensors and wearable sensors. The ambient sensors are fixed in a predetermined point of interest, and the wearable sensors are attached to the users.

#### 2.1.1. Ambient Sensors

A video camera is a typical ambient sensor used for human activity recognition, which makes use of computer vision techniques to recognize human activity from videos or images [7]. With the advance in sensing technology, a depth camera can capture the depth information in real-time, which inspired the research on activity recognition from 3D data [17].

Recent advances in the wireless community give solutions for activity recognition using a wireless signal. Studies have proved that the existence and movement of humans will affect the channel state information (CSI) of wireless signals [18,19]. CSI-based activity recognition has attracted numerous recent research efforts [18,19,20,21].

These ambient sensor-based methods are device-free and activity can be detected solely through the video/image or wireless signals. This advantage makes these methods especially suitable for security (e.g., intrusion detection) and interactive applications. The drawback of these methods is the predetermined infrastructures (video and WiFi devices) are difficult to attach to target individuals to obtain their data during daily living activities.

#### 2.1.2. Wearable Sensors

The limitation of ambient sensors motivated the use of wearable sensors for human activity recognition. The first wearable sensor-based activity recognition was proposed last 1990s, which used an accelerometer for posture and motion detection [22]. The accelerometer is the most frequently used sensor for activity recognition [23,24,25]. Attal et al. [26] present a review of different techniques used for physical human activity recognition from wearable inertial sensors data.

Lara and Labrador surveyed the state of the art in human activity recognition making use of wearable sensors [6].

With the advance of sensing technology, a smartphone is equipped with several sensors, including an accelerometer, gyroscope, magnetometer, and barometer. These sensors can be fused to recognize activity. Shoaib et al. proposed an activity recognition method by fusing smartphone motion sensors [27].

Compared with ambient sensor-based methods, wearable sensors are not limited by coverage, which can used for capturing people’s continuous activities.

### 2.2. Methods Used for Human Activity Recognition

#### 2.2.1. Traditional Machine Learning Methods

Traditional machine learning methods usually consist of two parts: feature extraction and classification. The activity recognition performance of the traditional methods depends highly on the extracted features. The feature extraction process requires laborious human intervention, which is the major challenge. The feature used for activity recognition includes time domain features (e.g., mean, standard deviation, variance, interquartile range, etc.), frequency domain features (e.g., Fourier Transform, Discrete Cosine Transform), and others (Principal Component Analysis, Linear Discriminant Analysis, Autoregressive Model) [6]. For activity classification, there are numerous classic methods, such as decision tree [23], Bayesian [28], neural networks [29], k-nearest neighbors [30], regression methods [31], support vector machines [32], and Markov models [33].

#### 2.2.2. Deep Learning Methods

Recently, deep learning has attracted considerable attention, and has been successfully applied in various fields, including human activity recognition. Zheng et al. [34] applied convnets to human activity recognition using sensor signals, but it just classified with a one-layer convolutional network which can hardly get high accuracy. Ronao and Cho proposed another deep learning method to classify six types of activities [35]. However, they use only accelerometers and magnetometers which can hardly differentiate vertical activities such as upstairs and downstairs. Gu et al. proposed a smartphone-based activity recognition method, which takes advantages of four types of sensor data, including an accelerometer, gyroscope, magnetometer, and barometer [36]. In their work, stacked denoising autoencoders is applied for activity recognition. The network just consists of two layers: encoding layer and decoding layer. The two-layer structure does not make full use of the no-linear advantage of deep learning, thus, leading to performance defects. Ravi et al. [37] propose a deep learning method for human activity recognition using low-power devices. To design a robust approach against transformations and variations in sensor properties, a manual feature selection strategy is adopted to extract features, which requires laborious human intervention.

## 3. Methodology

In this part, we will firstly introduce the activity categories for classification in detail, then give the experiment environment including software and hardware configuration. After that, the proposed CNN-based method is introduced as follows: the whole architecture of the CNN-based method, the data processing approach, the structure of our network, related theory and the vital training strategies. Finally, we are going to introduce the algorithm transplantation method which differs from the offline testing. Transplantation makes the online activity recognition with a Smartphone possible so that it can contribute to the indoor localization system.

### 3.1. Activities for Indoor Localization

In the indoor environment, human activity contains rich semantic information, which can be used for indoor localization. For example, if a user’s activity is detected as taking an elevator, the location can be inferred to the elevator. In this paper, we focus on the activities which contain context information. There are nine types of activities, including down elevator, down escalator, downstairs, up elevator, up escalator, upstairs, turning, walking, and still. The description of each activity is shown in Table 1.

### 3.2. Hardware and Software Setup

The specific software and hardware configuration information is given in Table 2. All our experiments were conducted on a server with powerful computational capabilities. There is 64-GB memory in the server and it is equipped with two GeForce GTX 1080Ti graphics cards to accelerate computing. We have installed Ubuntu 16.04 in junction with Python. Python has very efficient libraries for matrix multiplication, which is vital when working with deep neural networks. Tensorflow is a very efficient framework for us to implement our CNN architecture, moreover, we have to install the other dependencies like CUDA Toolkit and cudnn before using tensorflow. The CUDA Toolkit provides a comprehensive development environment for NVIDIA GPU accelerated computing. CuDNN can optimize CUDA to improve the performance.

### 3.3. Proposed CNN-Based Method

#### 3.3.1. Architecture

**Figure 1:**
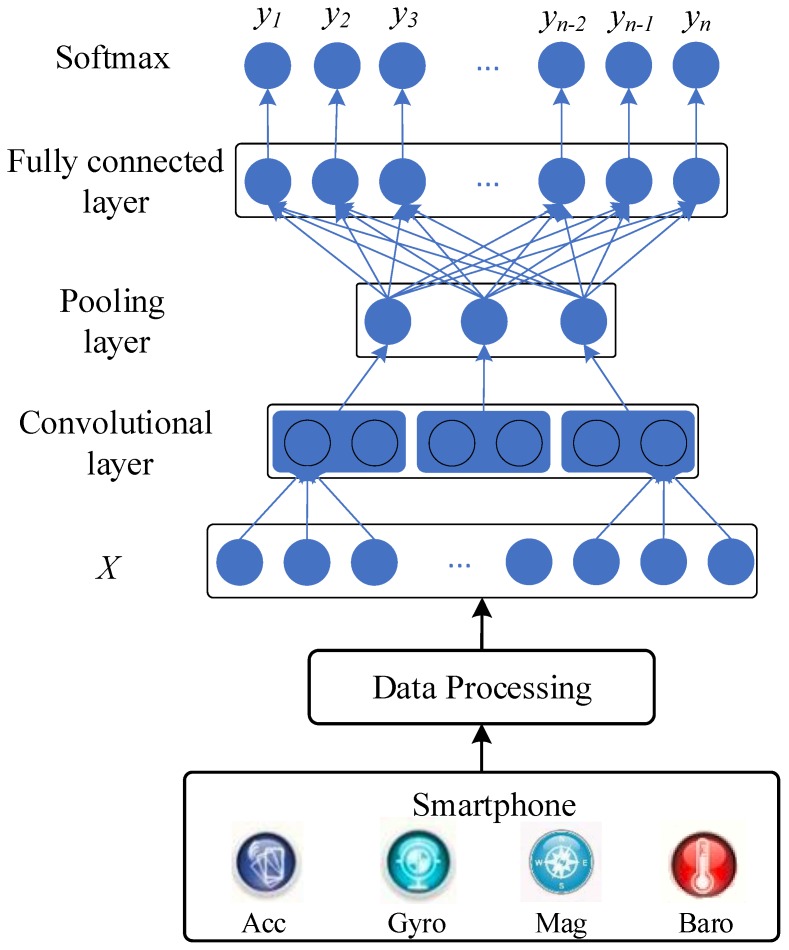
The architecture of the proposed method.

Figure 1 describes the architecture of our proposed method for activity recognition. Firstly, the activity data is collected by the built-in sensors of a smartphone, including an accelerometer, gyroscope, magnetometer, and barometer. Secondly, the collected data are divided to different segments. Each segment is an activity sample. Then, the data sample is put into the CNN for activity recognition.

#### 3.3.2. Data Segmentation

Data recorded is a time-varying signal which needs to be separated into training examples; each example stands for an activity. Sliding-window is used for segmentation and the window size stands for how long we take to present an activity. In activity recognition problem, the window size is of high importance. A too small value may not precisely capture the full characteristics of the activity, while a too large sequence may include more than one activity. To explore the proper window size we should use, we have chosen the varying window size of 1 s, 1.5 s, 2 s, 2.5 s, 3 s, 3.5 s with 50% overlap. For example, when the window size is 2 s, 1000 values recorded from the four sensors at each timestamp, we choose 1000 values to represent an activity.

#### 3.3.3. CNN for Activity Recognition

The structure of the convolutional neural network designed for activity classification mainly consists of three types of layers: convolutional layer, pooling layer and fully connected layer.

The input data is a vector containing data from an accelerometer, magnetometer, gyroscope and barometer, so 1D convolution is utilized to deal with the 1D data. If the input vector is $x=[x_{0},x_{1},\ldots ,x_{n-1}]$ (*n* is the number of data in a sliding window), the filter is written as $w=[w_{0},w_{1},\ldots ,w_{m-1}]$ (*m* is the filter size), and the output of the convolutional layer is $z=[z_{1},z_{2},\ldots ,z_{h}]$ (*h* is the length of the output vector), then for the *j*th element $z_{j}$ in *z*, it is satisfies the equation:

(1) $$z_{j}=\sum_{i=1}^{m-1}w_{i}x_{i+m*j}+b$$

where *s* refers to be the stride length of convolution. The relation between *h*, *m*, *n*, and *s* can be expressed as:

(2) $$h=\lceil \frac{n-m}{s}\rceil +1$$

After the convolutional layer, an extra activation layer is added to improve the express capability of the network, the output of activation layer is a=f(z), where *f* is the activation function. We use Relu as the activation function.

The pooling layer focuses on extracting more robust features by choosing the statistic value of nearby inputs. The maximum, minimum or mean value of a given region are computed as the output of the pooling layer, thus, reducing data shape, simplifying computing and avoiding overfitting. Max pooling is utilized in our proposed network [12].

After several layers of convolution and pooling operation, the fully connected layer follows as the classifier to recognize different activities. The softmax layer is applied to map the result of a fully connected layer to the range (0,1), which can be seen as the probability of the sample being each type of activity. Suppose the output of fully connected layer is $f=[f_{1},f_{2},\ldots ,f_{n}]$, the output of the softmax is $y^{\prime }=\left[y_{1}^{\prime },y_{2}^{\prime },\ldots ,y_{n}^{\prime }\right]$, it satisfies the equation that:

(3) $$y_{i}^{\prime }=\frac{e^{f_{i}}}{\sum_{k=1}^{n}e^{f_{k}}}$$

We adjust the weights and biases in each layer by minimizing the loss between the initial prediction and the label to get the best prediction model, the loss can be described as follows:

(4) $$h_{y^{\prime }}\left(y\right)=-\sum_{i}y_{i}^{\prime }log\left(y_{i}\right)$$

where *y* is the label of sample and $y^{\prime }$ is the output of the network. It is a least square problem which can be solved by some existing optimization algorithms like SGD, Momentum, RMSprop and Adam. We choose Adam as our optimization algorithm for the complexity of our network and the necessary of quick convergence.

#### 3.3.4. Training Strategies

The most time-consuming process of implementing activity recognition is training the model. There are two important tricks we have utilized in our classifier to accelerate training: Mini-batch Gradient Descent and normalization for the input data [38,39].

With the difference of the amount of training data, the optimization algorithm can be divided into Stochastic Gradient Descent, Mini-batch Gradient Descent and Batch Gradient Descent. The Stochastic Gradient Descent strategy just deals with one sample at a time while Batch Gradient Descent deals with all samples at a time. As one sample can hardly derive the right direction of gradient descent, the Stochastic Gradient Descent strategy always spends too much time to converge despite the high iteration speed. Batch Gradient Descent with low iteration efficiency is also not a good choice. Mini-batch Gradient Descent optimizing part of data is a proper strategy to filling the gap and we usually choose the power of 2 as the batch size.

Normalization of the input data can make the optimization easier and accelerate convergence. For the non-uniform distribution of input data in each dimension, there is much difficulty in finding the fastest gradient descent direction. Input data normalization can solve the problem by changing the distribution of the input data by this way: suppose an sample $x=[x_{1},x_{2},\ldots ,x_{m}]$ with mean μ and variance $\sigma^{2}$, where $\mu =\frac{1}{m}\sum_{i=1}^{m}x_{i}$, $\sigma^{2}=\frac{1}{m}\sum_{i=1}^{m}{\left(x_{i}-\mu \right)}^{2}$, we normalize the sample to

(5) $$\hat{x}=\frac{x-\mu }{\sqrt{\sigma^{2}+\varepsilon }}$$

where ε is a number close to zero for avoiding wrong division.

## 4. Experiments and Results

### 4.1. Dataset and Experimental Setup

For collecting the activity dataset, ten participants were invited to collect the data of nine activities defined in Table 1 using smartphones. The specific information of the participants is listed in Table 3. Data are collected from four types of sensors: an accelerometer, magnetometer, gyroscope and barometer. The data sampling rate is 50 Hz. 60% of the dataset are used to train and the rest 40% are used for the test data. The train and test data are from all the subjects. To avoid the effect of data dependence on the result, we shuffled the data in advance.

To evaluate the performance in identifying different types of activities, we choose the *F-value* as the evaluation metrics [40]. The *F-measure* is defined as F=(2∗P∗R)/(P+R) where *P* refers to the precision and *R* refers to the recall. In our experiments, the precision for an activity is the number of correctly expected samples (true positives) divided by the total number of samples predicted as belonging to this activity (the sum of true positives and false positives). The recall is defined as the number of corrected predicted samples divided by the total number of sample that actually belong to this activity (the sum of true positives and false negatives). Both of the precision and the recall rate can hardly measure the performance accurately. The *F-measure* is computed according to the precision and the recall, which can evaluate the performance more accurately.

### 4.2. Hyperparameter Settings

We have trained the data on network with different convolutional layers to find the best architecture. In each architecture, we adjust the filter size, number of feature maps, the pooling size, the learning rate and the batch size in the hyperparameters tuning process to retain the best configuration. As a result, we choose the best architecture with the best parameter setting as the final configuration. Table 4 illustrates the list of hyperparameters and their candidate values. The value in bold is the best setting of each hyperparameter.

### 4.3. Impact of Different Parameters

#### 4.3.1. Number of Layers

**Figure 2:**
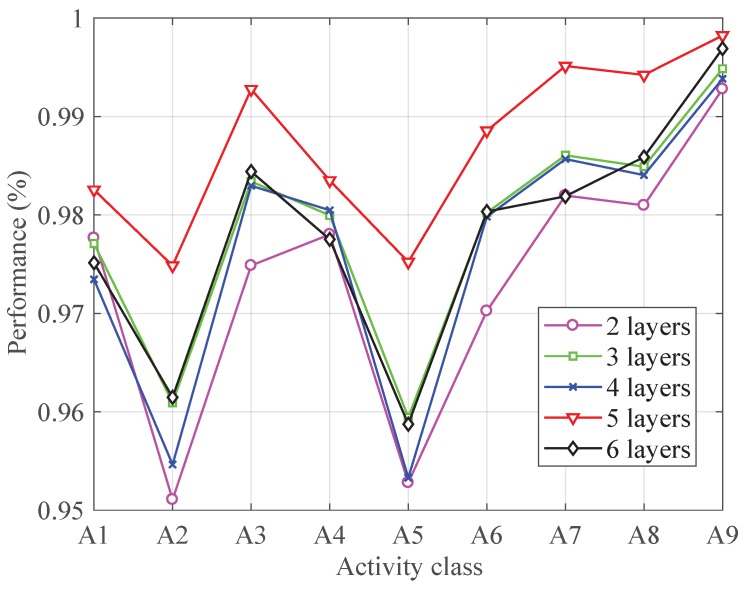
The performance with different convolutional layers.

To analyze the effect of number of layers, the CNN-based classifier was performed with different numbers of layers. Figure 2 shows the *F-measure* in each activity by using different number of layers. It is clear that the network with 5 convolutional layers outperforms others in each type of activities. The reason lies in the fact that network with convolutional layers less than 5 is not complex enough to extract appropriate features for activity recognition while network with six convolutional layers tends to cause over-fitting for the structure complexity. Network with five convolutional layers is just enough to obtain a good performance.

#### 4.3.2. Filter Size

**Figure 3:**
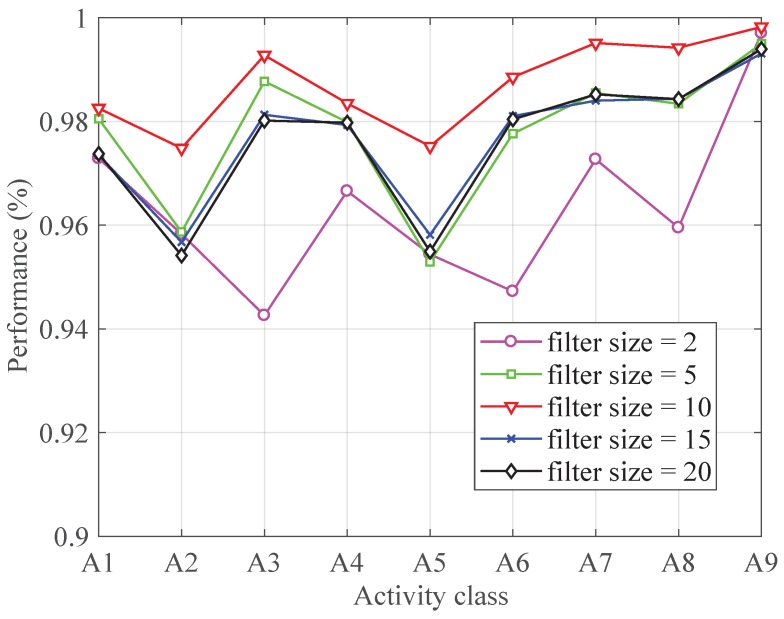
The performance with different filter size.

To obtain the most proper filter size, the classifier was performed with different filter size (the number of layer is set to 5). As shown in Figure 3, increasing the filter size from 2 to 10 improved the classification performance of each activity. When the filter size is larger than 10, the performance decreases with the increasing of filter size. Therefore, the filter size is set to 10.

#### 4.3.3. Number of Feature Maps

**Figure 4:**
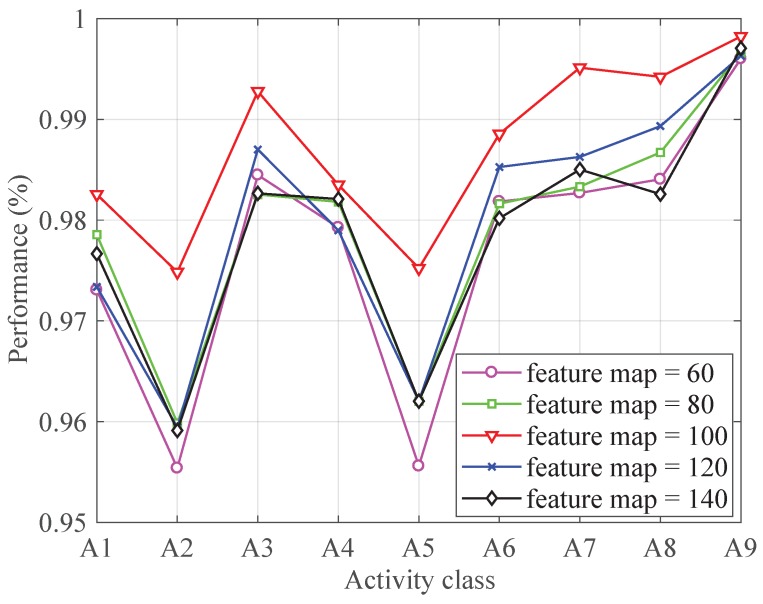
The performance with different feature map numbers.

Figure 4 shows the influence of the feature map on the classification performance. As shown in Figure 4, the best performance for each activity is achieved when the feature map is set to 100.

#### 4.3.4. Pooling Size

**Figure 5:**
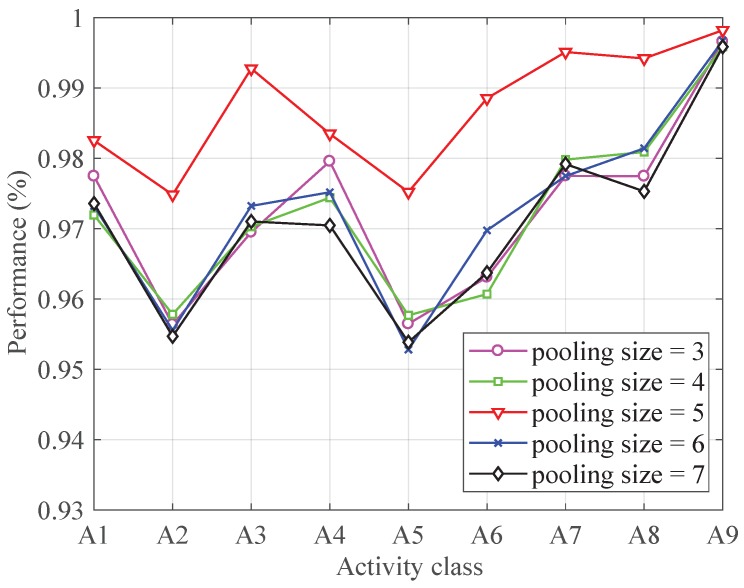
The performance with different pooling size.

Figure 5 demonstrates the classification performance for each activity with different pooling size. It can be seen from Figure 5 that the performance improves with the increasing of the pooling size at first. The classification achieves the best performance when the pooling size increases to 5. After that, the performance decreases with the increasing of the pooling size. Therefore, the pooling size is set to 5.

#### 4.3.5. Learning Rate

**Figure 6:**
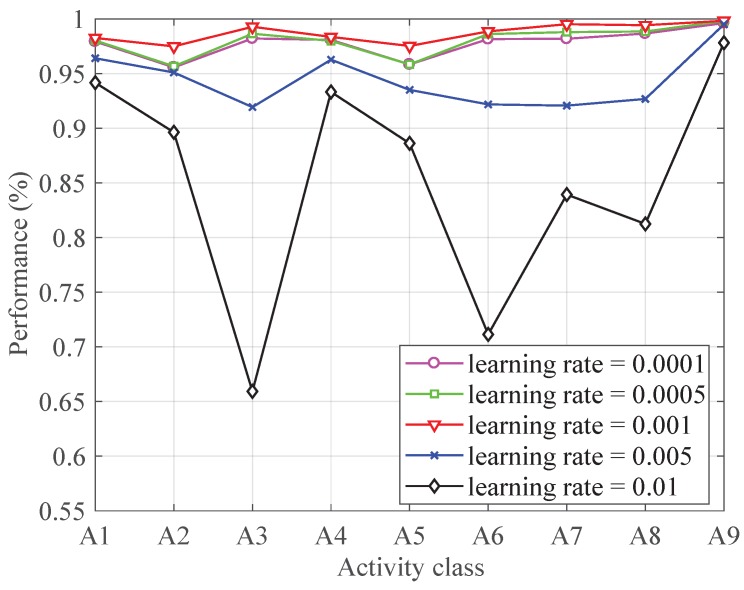
The performance with different learning rate.

Figure 6 shows the performance of each activity with different learning rate. It can be seen from Figure 6, when the learning rate is less than 0.001, the algorithm reaches steady and good performance while the learning rate bigger than 0.001 shows unstable results. The reason for the bad performance of big learning rate is that the variables update too fast to change to proper gradient descent direction timely. Though with good performance, the tiny learning rate is also not the best choice because it means the slow update of the variables and it leads to time consuming of the training. Therefore, the learning rate is set to 0.001.

#### 4.3.6. Batch Size

**Figure 7:**
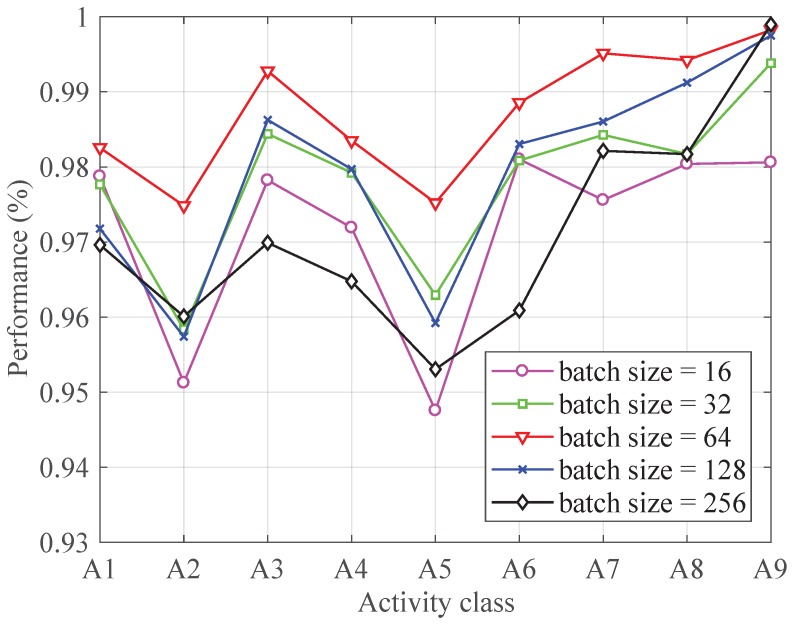
The performance with different batch size.

Figure 7 shows the performance of each activity with different batch size. The batch size is usually set to $2^{n}$. Here, we choose candidates of batch sizes from $2^{4}=16$ to $2^{9}=512$. In fact, the *F-measure* of each activity increases with the batch size increasing from 16 to 64 and it decreases with a batch size changing from 64 to 512.

### 4.4. Impact of Different Window Size

**Figure 8:**
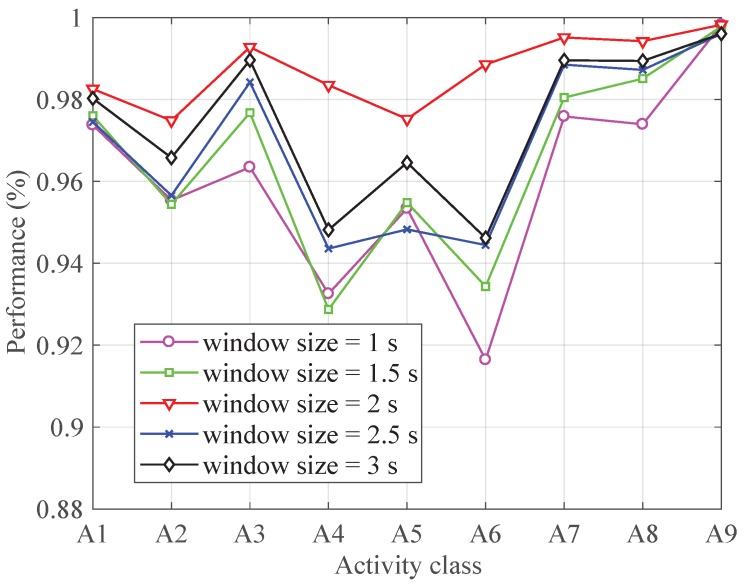
The performance with different window size.

During the data segmentation process, window size is a key factor. To evaluate the impact of window size on the classification performance, we divide the data in different window sizes, namely 1 s, 1.5 s, 2 s, 2.5 s, 3 s and 3.5 s. Figure 8 shows the performance of each activity with different window sizes. As can be seen from Figure 8, a window size of 2 s shows the best performance. Therefore, we choose 2 s as the window size for data segmentation.

### 4.5. Classification Performance

**Figure 9:**
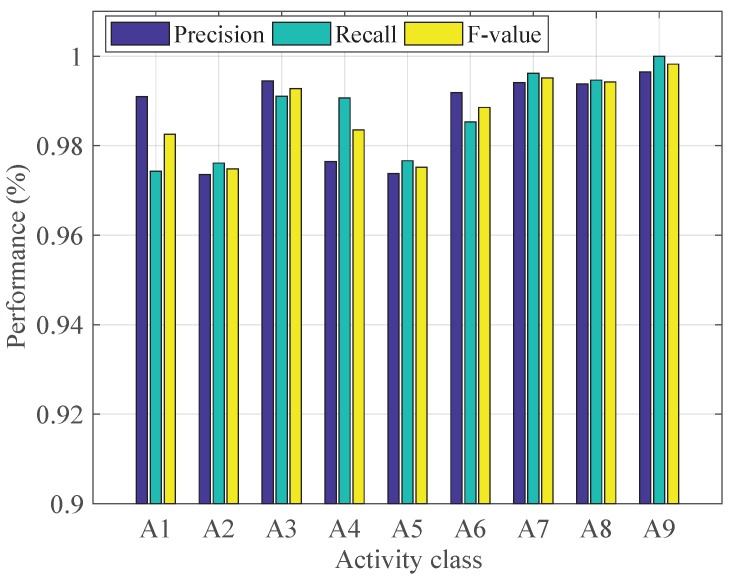
The performance for each type of activities with the best configuration.

Figure 9 shows the performance of our method in recognizing each activity. Table 5 shows the performance of the proposed method with the optimal hyperparmeters. From Figure 9, we can see that the proposed method shows excellent performance in recognizing all the nine activities. The *F-measure*s for all the activities are higher than 0.98 except A2 (down escalator) and A5 (up escalator), whose *F-measure*s are 0.97. Table 5 shows that there is a little difficulty for the proposed method to distinguish A2 and A5.

### 4.6. Comparison with Other Classification Methods

We have compared the proposed method with four traditional machine learning methods, including IBK, J48, NaiveBayes and SVM. The traditional machine learning methods need to select appropriate features. We use 64 features including statistical features, time domain features and frequency domain features (refer to [6]). Moreover, we have also compared the proposed method with another deep learning recognition approach named Convnet [35], which has a proven better performance than traditional ones like DT (decision tree) and SVM (Support vector machine). Convnet classified six types of human activities such as walking, upstairs, downstairs, standing, laying and sitting just utilizing an accelerometer and gyroscope.

**Figure 10:**
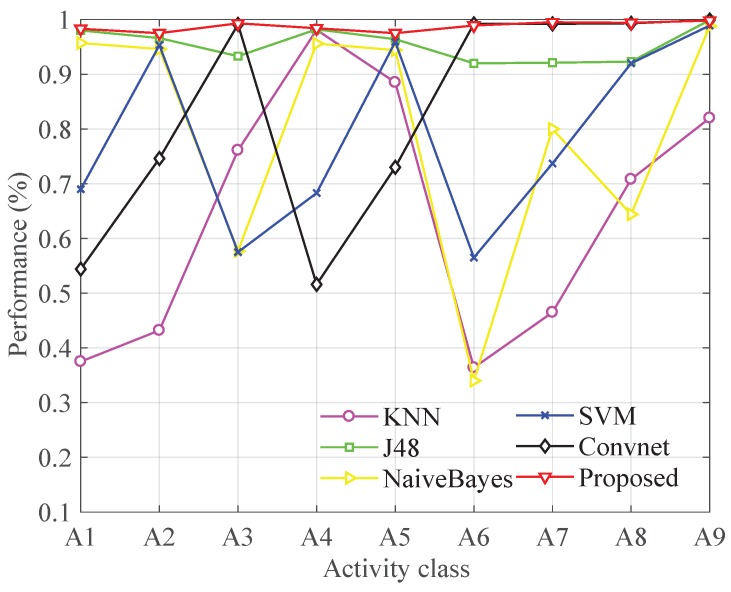
Performance comparison in each type of activity with other methods.

**Figure 11:**
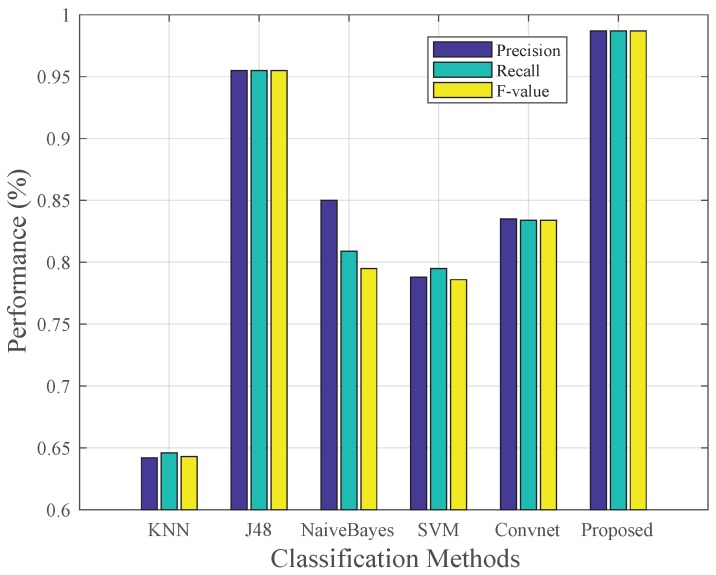
Comparison of average F-measure of nine activities with other methods.

Figure 10 and Figure 11 shows the comparison result. Figure 11 shows that the proposed method achieves 0.987 average *F-measure*s of nine types while J48 which performs best in other methods acquires the average *F-measure*s of 0.955. The other four methods show poorer performance with the *F-measure*s less than 0.85. Therefore, the proposed method performs better than state-of-the-art methods in dealing with indoor human activities. Figure 10 shows that only the proposed method and J48 can achieve relatively stable performance on every activity. However, J48 shows just 0.92 *F-measure*s on A6, A7 and A8 which is obviously worse than the proposed method. Furthermore, traditional machine learning methods require laborious human intervention in extracting complex hand-crafted features, so the J48 is time-consuming in the feature extraction process. Convet can hardly recognize similar activities such as up elevator and down elevator. We think the main reason is that Convnet is trained without barometer readings. We think that barometer is of high importance to sense the changing of altitude, so it can easily distinguish activities such as upstairs and downstairs which causes the change of height in different ways.

## 5. Conclusions

This paper presents a smartphone-based activity recognition method using a convolutional neural network, which can be used for indoor localization. The proposed method can learn the features automatically to avoid time-consuming feature extraction and selection. An activity database is built, which contains more than 6 GB of data collected by smartphones. Experimental results show that the proposed method achieves approximately 98% accuracy in identifying nine types of activities, which outperforms other classification methods.

There are some limitations in this work. Firstly, we did not consider the energy consumption problem in designing the recognition algorithm. Secondly, the dataset is collected from ten persons, which may not be enough to build a good model for the general population. In the future, we aim to keep collecting training data from more people. Meanwhile, we will investigate a deeper and more complex deep learning framework to improve the current method. Moreover, we will take into account the energy consumption in designing the recognition algorithm, for example, reducing the data sampling rate and the number of sensors used.

## Figures and Tables

**Table 1:** Types of activities to classify.

No.	Activity	Definition
A1	down elevator	Taking an elevator downward
A2	down escalator	Taking an escalator downward
A3	downstairs	Going down stairs
A4	up elevator	Taking an elevator upward
A5	up escalator	Taking an escalator upward
A6	upstairs	Going up stairs
A7	turning	Turn a corner
A8	walking	The user walks naturally
A9	still	The user remains static

**Table 2:** Types of activities to classify.

Software & Hardware	Configuration
CPU	Intel Xeon(R) CPU E5-2690 V4 @2.60 GHz × 28
Memory	64 GB
Graphics card	GeForce GTX 1080Ti× 2
CUDA	Cuda 8.0
cuDNN	Cudnn 6.0
GCC	Gcc 5.4.0
Python	Python 2.7
Tensorflow	Tensorflow 1.4.0

**Table 3:** The specific information of the participants.

No.	Height (cm)	Gender	Age
1	163	female	25
2	168	male	24
3	173	male	26
4	180	male	22
5	180	male	25
6	168	male	24
7	165	female	23
8	186	male	22
9	162	female	24
10	172	male	31

**Table 4:** List of hyperparmeters for the proposed CNN. The value in bold is the best setting of each hyperparameter.

Hyperparmeters	Description	Values
$N_{c}$	Number of convolutional layers	2, 3, 4, **5**, 6
$F_{s}$	Filter size	2, 5, **10**, 15, 20
$N_{m}$	Number of feature maps	60, 80, **100**, 120, 140
$P_{s}$	Pooling size	3, 4, **5**, 6, 7
$L_{r}$	Learning rate	0.0001, 0.0005, **0.001**, 0.005, 0.01
$B_{s}$	Batch size	16, 32, **64**, 128, 256, 512

**Table 5:** The confusion matrix of the proposed method

	Predicted	A1	A2	A3	A4	A5	A6	A7	A8	A9
Actual										
**A1**		3294	18	2	58	3	4	0	0	2
**A2**		15	3352	0	1	66	0	0	0	0
**A3**		4	1	3435	5	0	9	5	6	1
**A4**		7	3	2	3398	16	1	0	1	2
**A5**		2	65	0	11	3345	1	0	0	1
**A6**		1	3	9	6	4	3281	12	9	5
**A7**		0	0	5	0	0	3	3372	5	0
**A8**		1	1	1	1	1	9	3	3353	1
**A9**		0	0	0	0	0	0	0	0	3384
